# Sex differences and functional hemispheric asymmetries during number comparison

**DOI:** 10.1186/s13293-017-0162-6

**Published:** 2018-01-08

**Authors:** TiAnni Harris, Andrea Scheuringer, Belinda Pletzer

**Affiliations:** 1 Department of Psychology, Paris-Lodron University Salzburg, Hellbrunnerstrasse 34, A-5020 Salzburg, Austria; 2 Centre for Cognitive Neuroscience, Paris-Lodron University Salzburg, Hellbrunnerstr 34, A-5020 Salzburg, Austria

**Keywords:** Sex difference, Number magnitude comparison, Global advantage, Hemifield

## Abstract

**Background:**

Global-local stimuli are hierarchical structures consisting of a larger global structure which is composed of smaller local stimuli. Numbers are also constructed hierarchically, with multi-digit numbers being made up from single digits. During two-digit number comparison, compatible items (larger number contains larger unit digit, e.g., 53 vs. 68) are processed faster and more accurately than incompatible items (smaller number contains larger unit digit, e.g., 58 vs. 63). This so-called unit-decade-compatibility effect has challenged the holistic model of number processing and suggests that the processing of number magnitudes occurs at least in part, decomposed, i.e., separately for each digit. Thus, the compatibility effect is indicative of how decomposed numbers are processed, thereby sharing similarities with traditional global-local processing of hierarchical stimuli.

The goal of this study was to investigate whether factors that have been shown to reliably influence global-local processing also affect the compatibility effect during number comparison. Those include visual hemifield, sex, and menstrual cycle phase in women.

**Method:**

One hundred sixty participants, 77 naturally cycling women and 83 men, completed a two-digit number comparison task twice, with test-sessions time-locked to the early follicular or mid-luteal cycle phase in women. Number comparison stimuli were presented to the right or left hemifield, respectively.

**Results:**

We observed a stronger compatibility effect in the right visual hemifield compared to left visual hemifield and in women compared to men, but no evidence for an influence of menstrual cycle phase in women could be found.

**Conclusion:**

Hemispheric asymmetries in holistic versus decomposed number processing could be demonstrated for the first time, suggesting a similar hemispheric modulation for number magnitude processing as for global-local processing.

## Background

Global-local processing refers to the processing of stimulus hierarchies. In stimuli for which the global structure is made up of smaller local parts, the focus can either lie on the global structure or the local parts. The traditional task for studying global-local processing, i.e., hierarchical visual stimuli, was introduced by Navon [1]. Using these stimuli, Navon [1] discovered the global advantage effect, i.e., that responses are generally faster to global structures than to local parts [2–4]. Aside from certain stimulus characteristics (e.g., [2–5]), the global advantage effect is influenced by a number of individual differences, including sex and menstrual cycle phase, as well as hemispheric asymmetries and presentation mode (e.g., left or right hemifield, [6, 7]).

Sex differences have repeatedly been demonstrated for global-local processing using hierarchical stimuli, with stronger global processing in men and stronger local processing in women ([8–12]; but see [13]). Furthermore, in women, local processing in the Navon task is enhanced during the luteal phase of the menstrual cycle, i.e., when the female sex hormones estradiol and progesterone are both high [9].

However, the most reliable effect during global-local processing, concerns hemispheric asymmetries. Numerous visual hemifield studies suggest a right hemispheric preference for global and left hemispheric preferences for local processing (see [14] for a meta-analysis). Thus, the global advantage effect is higher when stimuli are presented to the right hemisphere (left hemifield presentation) than when presented to the left hemisphere (right hemifield presentation) [6, 7].

Hemispheric asymmetries have also been affected by sex in numerous tasks (see [15] for an overview), including the Navon task [10, 16, 17]. Specifically, hemispheric asymmetries in a variety of tasks are stronger in men compared to women (see [18] for a review). Additionally, menstrual cycle phase influences hemispheric asymmetries in a variety of tasks with stronger lateralization in low hormone phases, i.e., during menses and reduced lateralization in high hormone phases (see [18] for a review). These cycle-dependent changes in lateralization have been attributed to a reduction in inter-hemispheric inhibition by estradiol and progesterone [15, 18–20].

Several cognitive tasks rely on hierarchical stimulus material and may thus be influenced by global-local processing. This has recently been demonstrated for verbal and spatial stimulus material [21]. Another example is multi-digit number comparison. There is an ongoing debate whether the processing of multi-digit numbers is holistic or decomposed.

According to the holistic model, whole number magnitudes (e.g., 73) are processed by placing them on a logarithmically compressed mental number line [22, 23]. According to the decomposed model, each digit is processed separately (i.e., the decade 7 and the unit 3). Consistent with the holistic theory is the existence of a distance effect in number comparison, i.e., the comparison of numbers is performed faster the larger the distance between them [24–27]. Inconsistent with the holistic theory is the existence of the unit-decade compatibility effect, i.e., units influence reaction times, although they are not relevant for the comparison [26]. For example, the comparison between 53 and 68 (compatible item: 5 < 6 and 3 < 8) is performed faster than between 58 and 63 (incompatible item: 5 < 6 but 8 > 3). Therefore, some authors argue for a decomposed model of number comparison.

The unit-decade compatibility effect has been replicated in multiple studies (e.g., [26, 28, 29]) and can be utilized as a measure of decomposed number processing, i.e., the stronger the unit-decade compatibility effect, the more decomposed participants process two-digit numbers. A third model, the hybrid model [26, 30] suggests a combination of both theories, assuming that participants choose the most appropriate solution for the given situation automatically and individually, meaning the decomposed model is beneficial in certain situations for certain individuals, whereas the holistic model might work better for others. Accordingly, inter-individual differences in the unit-decade compatibility have already been observed.

For example, in a previous study Pletzer et al. [29] demonstrated a stronger compatibility effect in women than in men, suggesting that the sex of a person influences the performance of the number comparison task. Specifically, more decomposed processing was seen in women than in men. Since the structure of multi-digit numbers is hierarchical (whole number made up of single digits), they interpreted this finding as related to overall female tendency to process stimuli more strongly at a local level compared to men ([9–12]; but see [13]). Menstrual cycle phase did not influence the behavioral compatibility effect in the study of Pletzer et al., [29], but did influence the compatibility effect in brain activation.

If the unit-decade compatibility effect during number processing is indeed related to the overall tendency to process stimuli at a global or local level, the question arises whether hemispheric asymmetries, which strongly affect global-local processing, can also be observed for the unit-decade compatibility effect. Since the unit-decade compatibility effect is a measure of decomposed processing, a larger compatibility effect can be hypothesized, when number comparison stimuli are presented to the left hemisphere (right hemifield).

The aim of the present study was to investigate this question while controlling for other factors that have been shown to influence either the unit-decade compatibility effect or hemispheric asymmetries, i.e., sex and menstrual cycle phase.

To that end, we employed a two-digit number comparison task in a large sample of healthy young men and women. Stimuli are presented to the left or right visual hemifield. Furthermore, central presentation was used as a control condition in order to replicate sex and menstrual cycle influences irrespective of hemifield presentation. All participants completed the task twice with sessions in women being time-locked to the early follicular (low estradiol and progesterone) and the mid-luteal cycle phase (high estradiol and progesterone), respectively. Specifically, we hypothesize that:

(i) The compatibility effect is stronger with right hemifield presentation compared to left hemifield presentation.
(ii) Irrespective of their cycle phase, women show a stronger compatibility effect than men as observed in Pletzer et al. [29]. This hypothesis will also be tested in the control condition.
(iii) The hemispheric asymmetries in the compatibility effect are stronger in men compared to women, due to the previous findings of stronger lateralization in men during cognitive tasks [18].

Furthermore, we explore, whether in women the following:

(iv) A stronger compatibility effect in the luteal compared to the follicular phase can be observed with the larger sample size in the current study compared to the study of Pletzer et al. [29]. This hypothesis will also be tested in the control condition.
(v) Hemispheric asymmetries in the compatibility effect are stronger during the follicular compared to the luteal cycle phase, since previous findings demonstrated stronger hemispheric asymmetries in the follicular compared to the luteal phase during cognitive tasks [15].

## Methods

### Participants

The data presented in this manuscript were acquired as part of two larger studies. In total, participants were 77 healthy young women (mean age = 23.7, SD = 3.7) and 83 healthy young men (mean age = 24.6, SD = 4.0). Age did not differ significantly between men and women (*t*_(158)_ = 1.43, *p* = 0.15). All participants had completed their A-levels, were right-handed according to their self-report, had no diagnosis of psychological, neurological or endocrinological disorders, and were not currently on medication. All women had a natural menstrual cycle between 21 and 35 days of length with a mean duration of 29.41 days (SD = 3.02 days).

The study complied with the ethical standards as stated in the Declaration of Helsinki and was approved by the local ethics committee. Participants also signed informed consent, in which all requirements were listed and explained.

### Number comparison task

**Fig. 1:**
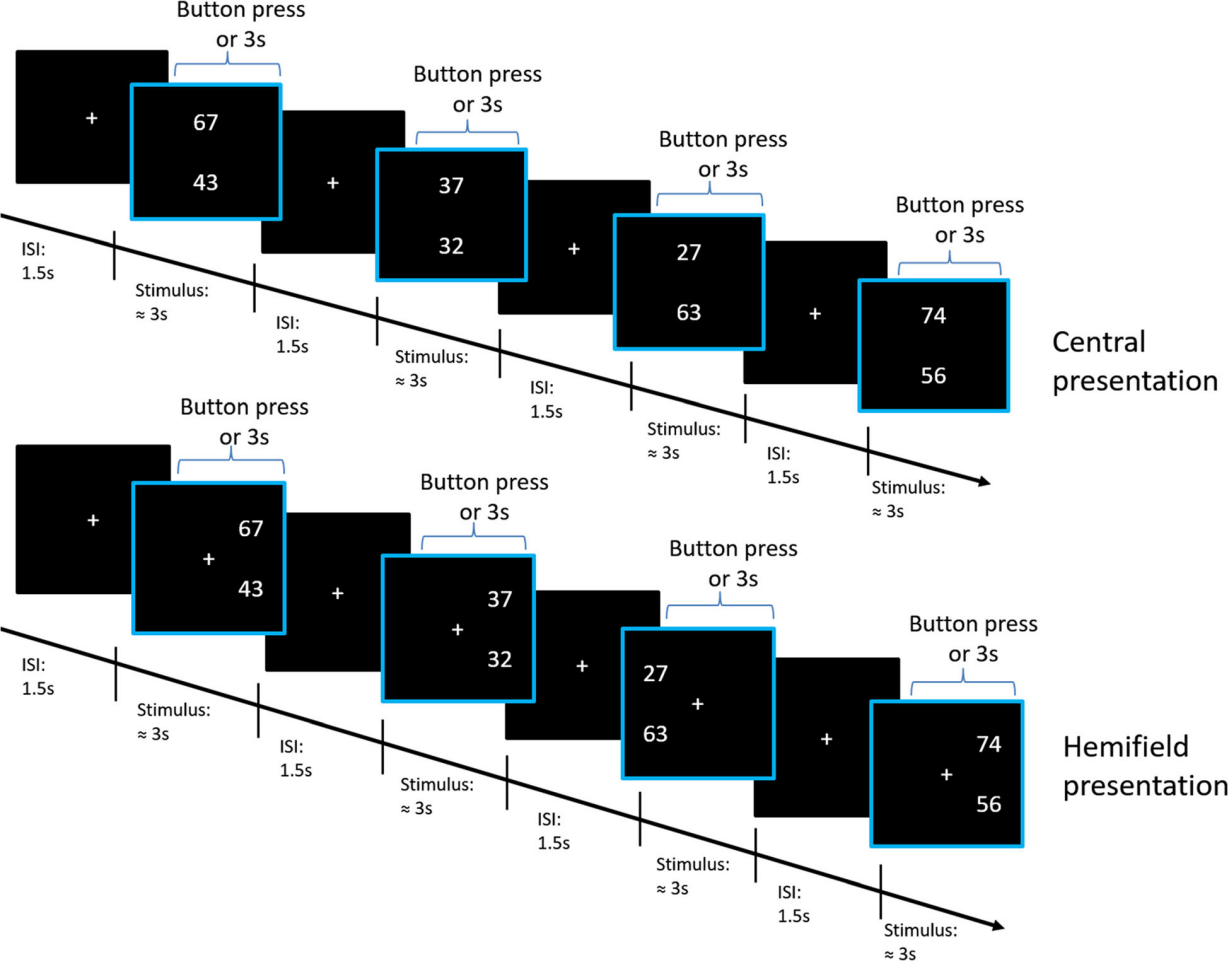
Central presentation (top) and hemifield presentation (bottom)

Two forms (A and B) of the number magnitude comparison task were created with the Presentation Software (version 0.71, 2009, Neurobehavioral Systems Inc., Albany, CA, USA). In each item, a pair of two two-digit numbers was presented in a vertical manner. Participants were seated at a fixed distance of 75 cm from the screen and had to identify the larger number by pressing either the left (for top number) or right (for bottom number) mouse button. At this distance, each number extended 2.29° of visual angle and the vertical distance between numbers was 6.49° of visual angle. Each item was presented for a maximum of 3 s and disappeared upon participant’s response. A fixation cross was presented for 1.5 s prior to each stimulus. Each form of the task was comprised of two conditions. A control condition included 100 number comparison stimuli, which were presented in the center of the screen. The hemifield condition included 200 number comparison stimuli, of which 100 each were randomly presented 6.84° of visual angle to the left or right of the fixation cross, respectively. In the hemifield condition, participants were instructed to always look at the fixation cross in the center of the screen and to not move their gaze, even if numbers appeared at the left or right side of the fixation cross. However, in the current setup of this laboratory, it was not possible to record saccadic eye-movements to control participant’s compliance with these instructions (see Fig. 1 for visual representation).

Among each set of 100 stimuli, 20 were within-decade pairs, in which both numbers contained the same decade digit (e.g., 57 vs. 52). They were included to ensure the relevance of unit digits for the comparison [19]. Of the remaining 80 stimuli, 40 were compatible pairs (e.g., 52 vs. 67) and 40 were incompatible pairs (e.g., 57 vs. 62). In compatible pairs, the larger number contained the larger unit-digit. In incompatible pairs, the larger number contained the smaller unit-digit. Half of the compatible and incompatible pairs, respectively, had a small (< 4) or large (≥ 4) decade distance. Problem size, overall distance, unit distance, units, decades, and parity were matched between compatible and incompatible items in each set, as well as between sets (see Table 1 for the most important parameters). It is not possible to match both decade distance and overall distance between compatible and incompatible items, but decade distance was matched between stimuli presented to the left and right hemifields in each set. Reaction times and accuracy were recorded for each item.

**Table 1:** Matching criteria for the number comparison task

	FormA				FormB			
	Left		Right		Left		Right	
	Comp	Incomp	Comp	Incomp	Comp	Incomp	Comp	Incomp
Upper number	58.83 ± 21.59	59.53 ± 20.59	58.95 ± 21.77	58.70 ± 21.94	59.28 ± 21.83	58.88 ± 23.89	58.58 ± 22.54	58.95 ± 20.90
Lower number	59.35 ± 21.55	59.45 ± 19.28	59.13 ± 23.54	59.83 ± 22.64	58.80 ± 23.24	59.23 ± 18.89	59.60 ± 23.76	58.40 ± 21.40
Larger number	75.80 ± 15.30	75.95 ± 14.33	75.83 ± 16.70	75.68 ± 15.16	75.83 ± 17.05	75.65 ± 16.37	75.80 ± 16.05	75.20 ± 15.50
Smaller number	42.38 ± 17.55	43.03 ± 14.74	42.25 ± 18.31	42.85 ± 14.56	42.25 ± 17.65	42.45 ± 16.14	42.38 ± 15.57	42.15 ± 15.50
Distance	33.43 ± 14.48	32.93 ± 14.21	33.58 ± 14.60	32.83 ± 16.89	33.58 ± 15.29	33.20 ± 16.36	33.43 ± 15.66	33.05 ± 16.05
Problem size (sum)	118.18 ± 29.09	118.98 ± 25.36	118.08 ± 31.86	118.53 ± 24.47	118.08 ± 31.16	118.10 ± 28.10	118.18 ± 27.48	117.35 ± 26.53
Decade distance	2.80 ± 1.48	3.83 ± 1.43	2.83 ± 1.51	3.80 ± 1.68	2.85 ± 1.58	3.85 ± 1.62	2.78 ± 1.61	3.80 ± 1.60
Unit distance	5.43 ± 1.45	5.33 ± 1.36	5.33 ± 1.26	5.18 ± 1.11	5.08 ± 1.18	5.30 ± 0.97	5.68 ± 1.33	4.95 ± 1.35

Scores are means ± SD

*Comp* compatible items, *Incomp* incompatible items, *FormA* first form of the number comparison task, *FormB* second form of the number comparison task, *decade distance* distance between the decades of the two numbers, *unit distance* distance between the units of the two numbers

### Procedure

Participants completed the number comparison task twice. The two test sessions were separated by approximately 2–3 weeks. The order of test forms across test-sessions was counter-balanced. Eighty-two participants (41 men, 41 women) completed Form A of the number comparison task during the first test session and Form B during the second test session. The remaining 78 participants (42 men, 36 women) completed Form B of the number comparison task during the first test session and Form A during the second test session.

In women, test-sessions were time-locked to the early-follicular and mid-luteal cycle phase, respectively. The early follicular phase started with the onset of menses and lasted up to 5 days before ovulation. The mid-luteal phase included days 3–10 after ovulation. Scheduling of luteal test sessions was performed as follows. Participants self-reported the onset of their last menstruation and usual cycle duration. Based on this information, the expected onset of the next menstruation was calculated. The date of ovulation was then calculated as 14 days before the expected onset of the next menstruation and test sessions were scheduled 3–10 days after that date. The date of ovulation was confirmed by commercial ovulation tests (Pregnafix®, ATT Drogerievertriebs GmbH, Salzburg), which indicate the LH surge in urine. Furthermore, the onset of next menstruation was confirmed by follow-up reports. Nevertheless, in five women, follow-up reports indicated that the onset of next menses was not around the expected date, suggesting that both test sessions took place in the same cycle phase. These women were excluded from menstrual cycle analyses. The order of test sessions and test forms across cycle phases was counter-balanced. Of the remaining 72 women, 36 women completed the first test session (20 Form A, 16 Form B) during the early follicular phase and the second test session during the mid-luteal phase. The other 36 women completed the first test session (20 Form A, 16 Form B) during the mid-luteal cycle phase and the second test session during the early follicular phase. Thus, 36 women completed Form A during their follicular phase and Form B during their luteal phase. The remaining 36 women completed Form A during their luteal phase and Form B during their follicular phase.

### Statistical analyses

Data were analyzed using statistics software R 3.3.2.

In a first step, a positive compatibility effect was confirmed for each group (men/women), session, and condition (left/right hemifields). To that end, reaction times of each group and condition were analyzed in the context of linear mixed effects models (lmes) using the *lme* function of *nlme (3.1–131)* package. Accuracy of each group and condition was analyzed in the context of a generalized linear mixed effects model (glme) using the glme function of the lme4 package (1.1–13). Only reaction times to correctly solved items were considered. Participant number was modeled as a random factor and compatibility as a fixed effect (formula: e.g., RT ~ 1|PNr + compatibility).

In a second step, the effect size of the compatibility effect in RT was calculated for each participant and hemifield as standardized mean difference [31] between RT to incompatible and compatible items, such that a positive compatibility effect reflects slower reactions to incompatible compared to compatible items. Thereby, variations in response times between items are taken into account. Only reaction times to correctly solved items were considered. The compatibility effect in accuracy was calculated as difference in accuracy between compatible and incompatible items, such that a positive compatibility effect reflects lower accuracy in incompatible compared to compatible items. Thus, for both compatibility measures, a larger compatibility effect reflects more decomposed number processing. The compatibility effects for both RT and accuracy were analyzed in the context of lmes. In all models, participant number was modeled as a random factor to control for repeated measurement. In order to control for learning effects, session was included as fixed effect in all models. For hemifield presentation, we modeled “hemifield” and its interaction with “sex” in the total sample, as well as its interaction with “cycle phase” in the female sample. Since menstrual cycle effects did not reach significance, effects of sex were not evaluated separately for the follicular and luteal cycle phase. The effects of sex and cycle phase were additionally also assessed in lmes over the compatibility effects during central presentation for comparison with our previous data. All model details including formulas are included in the respective paragraphs of the results section.

In all models, both the dependent and continuous independent variables were *z*-standardized using the *scale* function. Therefore, the coefficient *b* of fixed effects in the models represent a standardized effect size based on standard deviations, similar to Cohen’s *d*.

## Results

A positive compatibility effect was observed in all groups and conditions (for RT: all b > 0.16, all SE_b_ < 0.01, all *t* > 12.88, all *p* < 0.001; for accuracy: all b > 3.08, all SE_b_ < 0.09, all Z > 24.00, all *p* < 0.001 compare Table 2).

**Table 2:** Accuracy and reaction times for compatible and incompatible items in all groups and conditions

			Accuracy in %		Reaction time in ms	
		Hemisphere	Incompatible	Compatible	Incompatible	Compatible
Women	Session 1	Left	92.71	96.79	875.14	836.80
		Right	93.31	96.23	875.48	827.12
		Central	92.88	97.25	776.30	722.69
	Session 2	Left	91.61	96.06	807.15	767.76
		Right	92.24	96.62	809.63	760.03
		Central	91.87	96.87	726.47	673.28
Men	Session 1	Left	94.48	97.86	791.81	760.38
		Right	94.21	97.29	797.28	762.25
		Central	93.25	97.62	693.07	652.23
	Session 2	Left	94.09	97.43	771.93	733.88
		Right	93.61	97.25	780.30	739.94
		Central	92.62	97.81	669.36	624.09

### Effect of sex and hemifield on the compatibility effect

Since no menstrual cycle dependent changes (see below) were observed, menstrual cycle phase was not controlled in the analysis of sex differences. The compatibility effects in accuracy and reaction time were each analyzed in the context of an lme. These included participant number as a random factor as well as session and the effects of sex and hemifield and their interaction as fixed effects (formula: compatibility ~ 1|PNr + session + hemifield*sex) in order to evaluate whether

(i) The compatibility effect is stronger with right hemifield presentation compared to left hemifield presentation [main effect of hemifield].
(ii) Irrespective of their cycle phase, women show a stronger compatibility effect than men as observed in Pletzer et al. [29] [main effect of sex].
(iii) The hemispheric asymmetries in the compatibility effect are stronger in men compared to women [interactive effect of hemifield*sex].

**Fig. 2:**
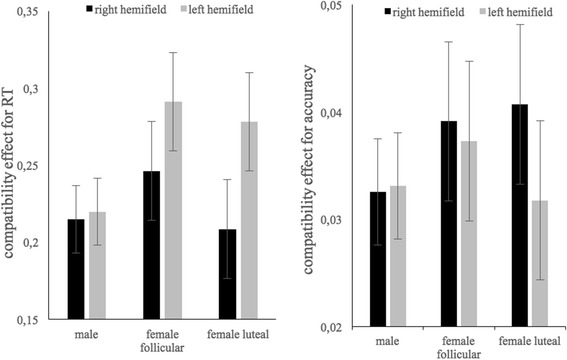
Standardized compatibility effect for accuracy and reaction times for male and females in follicular and luteal phase for left and right hemifields (CIs = 1 SE)

For the compatibility effect in reaction time, we observed significant main effects of session (*b* = 0.16, SE_*b*_ = 0.07, *t*_(477)_ = 2.31, *p* = 0.022), hemifield (*b* = −0.24, SE_*b*_ = 0.10, *t*_(477)_ = −2.47, *p* = 0.014) and sex (*b* = −0.28, SE_b_ = 0.12, *t*_(158)_ = −2.19, *p* = 0.030). The compatibility effect was larger for the first session compared to the second session, for the right hemifield compared to the left hemifield, and for women compared to men. The interaction between sex and hemifield did not reach significance (*b* = 0.22, SE_*b*_ = 0.14, *t*_(477)_ = 1.62, *p* = 0.104) (Fig. 2). For the compatibility effect in accuracy, no significant effects of session, hemifield or sex and no significant interaction between sex and hemifield were observed (all *b* < 0.08, SE_*b*_ < 0.12, *t*_(477)_ < 1.45, *p* > 0.15).

### Menstrual cycle-dependent changes in the compatibility effect

For the female sample, additional lmes were run for the compatibility effects in accuracy and RT. They included participant number as a random factor as well as session and the effects of hemifield and cycle and their interaction as fixed effects (formula: compatibility ~ 1|PNr + session + hemifield*cycle) in order to evaluate, whether

(iv) Women show a stronger compatibility effect during luteal phase than during follicular phase [main effect of menstrual cycle]
(v) The hemispheric asymmetries in the compatibility effect are stronger in follicular cycle phase compared to luteal cycle phase [interactive effect of menstrual cycle*hemifield]

For the compatibility effect in RT a significant main effect of session was observed (*b* = 0.20, SE_*b*_ = 0.10, *t*_(212)_ = 1.99, *p* = 0.05). Furthermore, no significant main effect for hemifield or cycle and no significant interaction between cycle and hemifield were observed for the compatibility effect in both accuracy and reaction time (all *b* < 0.12, SE_*b*_ > 0.08, *t*_(212)_ < 1.32, *p* > 0.18).

For the compatibility effect in accuracy, no significant effects of session, hemifield, or sex and no significant interaction between sex and hemifield were observed (all *b* < 0.12, SE_b_ > 0.08, *t*_(212)_ < 1.32, *p* > 0.18).

Central presentation was used as a control condition in order to replicate sex and menstrual cycle influences irrespective of hemifield presentation. For central presentation, neither session, nor sex, or menstrual cycle phases did affect the compatibility effect in RT or accuracy (all *b* < 0.13, all SE_*b*_ < 0.09, all *t* < 1.41, all *p* > 0.16).

## Discussion

The goal of the present study was to evaluate hemispheric asymmetries in the compatibility effect during number comparison while controlling for the impact of sex and menstrual cycle phase. According to the proposed similarity between the compatibility effect and local processing, we hypothesized a higher compatibility effect (i) in the right compared to the left hemifield, (ii) in women compared to men, (iv) as well as during the luteal cycle phase compared to the follicular cycle phase. We furthermore hypothesized that (iii) hemispheric asymmetries in the compatibility effect would be stronger in men compared to women and (v) in the follicular cycle phase compared to the luteal cycle phase.

Regarding hemispheric asymmetries, the hypothesis that the compatibility effect is stronger with right hemifield presentation (left hemisphere) compared to left hemifield presentation (right hemisphere) was confirmed for reaction times (RT). This suggests a stronger specialization of the left hemisphere for decomposed number processing. Since a left-hemispheric dominance has previously been reported for local processing [32], this finding supports the idea, that the compatibility effect is related to the overall tendency to process stimuli on a local level [9, 29].

Regarding sex differences, the hypothesis of a larger compatibility effect in women compared to men was also confirmed for RT. This supports the notion of more holistic number processing in men, but more detail-oriented number processing in women. Comparably, a stronger compatibility effect in RT in women compared to men was previously reported for central presentation [29]. However, in the present study, the compatibility effect with central presentation did not differ between men and women. One speculative reason for this discrepancy may be that the vertical distance between the numbers as presented in the present study was much smaller than in our previous study. We were previously able to demonstrate that the compatibility in men depends on the vertical distance between numbers, such that a larger compatibility effect was found with a larger distance between numbers [33]. If numbers are presented closely together, men are more likely to process them in a decomposed manner than when they are presented farther apart. Thus, in the present study with the smaller vertical distance, men may have processed numbers more decomposed than in the previous study, thus reducing the sex difference in the compatibility effect with central presentation. The vertical distance between numbers may play a smaller role for the compatibility effect when not presented in the fovea, as is the case with hemifield presentation.

Regarding menstrual cycle effects, no significant changes in the compatibility effect were observed between the follicular and luteal phase of the menstrual cycle. This is in line with previous behavioral results of Pletzer et al. [29], though sample size in the present study was much larger. While menstrual cycle dependent changes have previously been observed for some measures of global-local processing, like the global advantage effect in the Navon paradigm [9], global-local processing in other tasks, e.g., the Kimchi-Palmer task [12], were not affected by menstrual cycle phase. Accordingly, not all measures of global-local processing might be equally sensitive to menstrual cycle-dependent changes.

Furthermore, in the present study, neither sex nor menstrual cycle phase did interact with the hemispheric asymmetries in the compatibility effect. The exploratory hypotheses of stronger hemispheric asymmetries in men compared to women, and during the follicular compared to the luteal cycle phase could not be confirmed. However, our study differs from previous studies on lateralization in that it does not assess how strongly a whole task is lateralized to the left or right (compare [18] for a review). Rather, we investigate how much different strategies in the same task differ in their lateralization to the left or right.

It is also noteworthy that both effects confirmed in the present study, i.e., the hemifield effect and sex differences in the compatibility effect, were confirmed for RT not accuracy. Also in our previous studies, the compatibility effect in RT was more sensitive to task-related factors [33] or sex differences [29].

One limitation of the present study is that saccadic eye movements could not be controlled for. This may have negatively affected the hemifield results, such that hemispheric asymmetries were not detectable for the compatibility effect in accuracy.

## Conclusion

In summary, we observed a stronger compatibility effect in the right visual hemifield (left hemisphere) than in the left visual hemifield (right hemisphere) and in women compared to men, but no evidence was found for an effect of menstrual cycle. This is the first study demonstrating hemispheric asymmetries in holistic vs. decomposed number processing. The results suggest a hemispheric modulation of number magnitude processing similar to global-local processing.

## Abbreviations

Comp: Compatible items
Decade distance: Distance between the decades of the two numbers
FormA: First form of the number comparison task
FormB: Second form of the number comparison task
Incomp: Incompatible items
RT: Reaction time
Unit distance: Distance between the units of the two numbers
